# P43 - Psycho-social impact of parenting children with food allergy

**DOI:** 10.1186/2045-7022-4-S1-P98

**Published:** 2014-03-14

**Authors:** Aaron Cortes, Alicia Sciaraffia, Angela Castillo

**Affiliations:** 1 Universidad de Chile Clinical Hospital, Santiago, Chile

## 

Raising children with chronic diseases has been described as a stressing situation for carers. Several studies, especially in asthma and rhinitis, have documented psychological and physiologic impact on carers. However, little work describing the psychosocial impact of raising children with food allergy (FA) has been done.

Consecutive carers (63) from the Universidad de Chile Clinical Hospital and from a peer support group for parents (Creciendo con Alergias) were recruited and evaluated for anxiety, depression, perceived social support and personality. Carers´ reaction due to children´s symptoms was evaluated using a scale developed by the authors.

In terms of personality characteristics 40.3% of carers were anankastic, 22.6% were histrionic and 12.3% were dependant. Psychologically, 44.4% had anxiety, 15.9% had depression and 40% perceived social support as poor. Retarded allergic reaction correlated with higher carers’ irritability (r = .343; p< .01), while immediate reactions correlated with higher carers’ anxiety (r = -.311, p< .05). A higher number of gastric symptoms correlated with: poorer carers’ management of crises (r = -.576; p< .01); perception of been psychologically affected by child’s symptoms (r = .455; p< .01); and perception of intensification of symptoms due to family crises (r = .442; p< .01). Runny nose was associated with lower carer’s interaction with their friends (r = -.311; p< .05) and abdominal pain correlated with poor management of crises (r = .499; p< .01).

Parenting children with FA is a stressing situation that affects carers’ psychological balance and social interaction. The level of symptoms also affects carers’ perception of their ability to deal with crises, which is perceived as a factor able to intensify children’s symptoms; a vicious circle that exacerbate the negative impact of raising a child with FA. A personality profile was described; however, this needs further study to be properly interpreted.

